# MORTALITY PROFILE FROM PEDIATRIC BONE NEOPLASIA IN MINAS GERAIS (2015–2024)

**DOI:** 10.1590/1413-785220263405e300018

**Published:** 2026-08-10

**Authors:** Alvaro Inacio de Moura, Rafael Moreno Chaves, Fhilipe Hollanda Cavalcanti Soares

**Affiliations:** 1 Associacao Evangelica Beneficente de Minas Gerais, Belo Horizonte, MG, Brazil.

**Keywords:** Bone neoplasms, Pediatrics, Mortality, Epidemiology, Neoplasias ósseas, Pediatria, Mortalidade, Epidemiologia

## Abstract

**Objectives:**

To analyze the profile of deaths from malignant neoplasms of bones and articular cartilage in the pediatric population of Minas Gerais (2015–2024), considering sociodemographic, clinical, epidemiological, and regional aspects.

**Methods:**

Epidemiological, documentary, descriptive, and retrospective study with a census design, including deaths of individuals aged 0–19 years residing in Minas Gerais (ICD-10 C40–C41) registered in SIM/DATASUS. Variables analyzed included sex, age, race/color, municipality, place of death, length of hospital stay, and hospital costs. Descriptive statistics were used with frequencies, percentages, and mortality rates.

**Results:**

There were 3,883 hospitalizations and 79 deaths, predominating male patients (57.7% of hospitalizations; 59.5% of deaths), adolescents aged 10–19 years, and individuals of mixed race/color (pardo). Belo Horizonte accounted for most hospitalizations and deaths, while Juiz de Fora had high hospitalization rates but low mortality. Longer hospital stays were observed in female patients and those under 1 year; the highest costs were among adolescents aged 10–14 years. Hospitalizations decreased over time, but mortality remained stable.

**Conclusions:**

Findings highlight the need for early detection, access to specialized therapies, and reduction of regional inequalities to improve clinical outcomes and equity in pediatric oncology care. ***Level of Evidence IV; Retrospective cohort study.***

## RESUMO

**Objetivos:**

Analisar o perfil dos óbitos por neoplasias malignas dos ossos e cartilagens articulares na população pediátrica de Minas Gerais (2015–2024), considerando aspectos sociodemográficos, clínicos, epidemiológicos e regionais.

**Métodos:**

Estudo epidemiológico, documental, descritivo e retrospectivo, com delineamento censitário, incluindo óbitos de 0 a 19 anos residentes em Minas Gerais (CID-10 C40–C41) registrados no SIM/DATASUS. Variáveis analisadas: sexo, idade, cor/raça, município, local de óbito, tempo de internação e custos hospitalares. A análise foi descritiva, com frequências, percentuais e taxas de mortalidade.

**Resultados:**

Foram registradas 3.883 internações e 79 óbitos, predominando pacientes masculinos (57,7% das internações; 59,5% dos óbitos), adolescentes de 10 a 19 anos e pardos. Belo Horizonte concentrou mais internações e óbitos; Juiz de Fora apresentou alta hospitalização com baixa mortalidade. Maior permanência hospitalar entre pacientes do sexo feminino e menores de 1 ano; custos mais elevados entre 10 e 14 anos. As internações reduziram ao longo do período, mas a mortalidade manteve-se estável.

**Conclusão:**

Evidencia-se a necessidade de detecção precoce, de acesso a terapias especializadas e de redução das desigualdades regionais para melhorar os desfechos clínicos e a equidade no cuidado oncológico pediátrico. ***Nível de Evidência IV; Estudo de coorte retrospectivo.***

## INTRODUCTION

Neoplasia is characterized by abnormal and uncontrolled cell growth. When it exceeds the limits of the site of origin and invades neighboring tissues or spreads to other regions of the body, it is classified as malignant. Unlike normal cells, neoplastic cells do not follow the expected life cycle: they continue to proliferate and can spread both locally and through the bloodstream^1^.

In bones and articular cartilages, malignant tumors can arise primarily, when they originate in the tissue itself, or secondarily, through metastases from other organs. These cancers present with heterogeneous clinical manifestations that directly influence prognosis. Although infrequent, they occur more frequently in children and adolescents^2^.

The musculoskeletal system is composed of specialized tissues with complementary functions. Bone, rigid, vascularized, and metabolically active, serves in body support, organ protection, locomotion, and mineral storage, in addition to having a great capacity for remodeling^3^. In contrast, cartilage, flexible and poorly regenerative, covers joints to reduce friction and absorb impacts^4^. Both can be affected by neoplasms, such as chondrosarcoma in cartilage and, especially in young people, osteosarcoma and Ewing's sarcoma^3-4^.

Malignant bone neoplasms in childhood are rare, accounting for 3%–5% of cancers in individuals under 15 years old. Among them, osteosarcoma stands out as the most prevalent (3.8/million), preferentially affecting the metaphyses of long bones, and Ewing's sarcoma (3.5/million), characteristic of adolescence, often located in the pelvis and diaphyses^5-7^.

The initial recognition of the disease usually occurs after consultation with a pediatrician, general practitioner, or orthopedic surgeon. Warning signs include progressive pain, with variable intensity depending on the tumor stage, and an increase in volume in the affected area, noticeable as the disease progresses^8^.

The definitive diagnosis is based on imaging tests. X-ray is the initial method due to its wide availability, but it has limitations. CT and MRI allow for detailing the extent and tumor invasion, while advanced techniques, such as PET-CT, contribute to metabolic assessment and therapeutic planning^9^.

The treatment is necessarily multimodal. It involves chemotherapy to reduce the tumor and control micrometastases, surgery for resection with limb preservation whenever possible, and, in specific situations, radiation therapy, especially in Ewing's sarcoma. Thanks to advances in surgical techniques and chemotherapy protocols, patients’ survival and quality of life have improved significantly. Still, the prognosis continues to depend on the tumor type, disease stage, and initial response to therapy^10^.

In this context, it is observed that deaths from malignant neoplasms of bones and articular cartilages in the pediatric population pose a significant challenge for public health, both due to clinical and social impact. These diseases, although infrequent, present high aggressiveness, potentially progressing rapidly to severe outcomes, including fatal ones. Factors such as age group, presence of comorbidities, and advanced stage at diagnosis increase patients’ vulnerability to worse outcomes. In this context, analyzing the profile of deaths is essential to support strategies for early detection, expand access to specialized therapies, and improve the quality of care, thereby contributing to reduced mortality and the sustainability of the healthcare system.

## OBJECTIVES

The general objective of this study is to analyze the profile of deaths from malignant neoplasms of the bones and articular cartilages among the pediatric population in the state of Minas Gerais, from 2015 to 2024. Specifically, the aim is to describe the distribution of deaths according to sociodemographic variables such as age, sex, and municipality of residence; characterize the deaths concerning clinical and epidemiological variables, including length of hospitalization, location of occurrence, and presence of associated comorbidities; evaluate the temporal trend of mortality due to malignant neoplasms of the bones and articular cartilages during the studied period; compare the profile of deaths according to age groups of the pediatric population (0–4 years, 5–9 years, 10–14 years, and 15–19 years); and identify possible differences in outcomes according to the health region of the state, aiming to understand inequalities in pediatric oncological care.

## METHODOLOGY

This was an epidemiological, documentary, quantitative, descriptive, and retrospective study, with a census design that encompassed all deaths among children and adolescents resulting from malignant neoplasms of the bones and articular cartilages recorded in the state of Minas Gerais from January 1, 2015, to December 31, 2024. The data were obtained through the Mortality Information System (SIM) and the public databases of the Department of Informatics of the Unified Health System (DATASUS).

All cases of death in individuals aged 0 to 19 years, residing in Minas Gerais, whose underlying cause was classified under codes C40 and C41 of the International Classification of Diseases, 10th revision (ICD-10), were included. Records concerning non-residents of Minas Gerais, fetal deaths, and those with inconsistent or incomplete information that hindered analysis were excluded.

Data collection was conducted by consulting the public databases of SIM/DATASUS, using the TabNet tool to extract the available information. The variables analyzed included year of death, sex, age group, race/color, municipality of residence, location of death, and ICD-10 subcategories.

The data were organized and tabulated in electronic spreadsheets using Microsoft Excel 2020. The analysis was conducted using descriptive statistics, presenting absolute numbers, relative frequencies, percentages, and mortality rates, and preparing tables and graphs to facilitate visualization of the results. The temporal and geographical distribution of deaths was evaluated, as well as the sociodemographic profile of the affected pediatric population in the state throughout the studied period.

## RESULTS

The analysis of hospitalizations for malignant neoplasms of the bones and articular cartilage among the pediatric population of the state of Minas Gerais from 2015 to 2024 revealed a total of 3,883 records.

**Figure 1:**
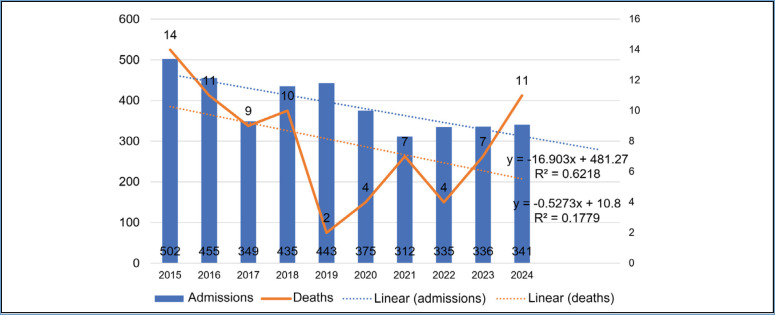
Distribution of the profile of hospitalizations/deaths of patients with malignant neoplasms of the bones and articular cartilage in the pediatric population of the state of Minas Gerais, 2015 to 2024. (hospitalizations=3,883; deaths=79).

Source: DATASUS-SIH.

The joint analysis of hospitalizations and deaths due to malignant neoplasms of the bones shows annual variations, but with a general trend of reduction in the number of hospitalizations over the historical series. In 2015, 502 hospitalizations were recorded, a number that fell to 341 in 2024, representing a decrease of approximately 32.1%. Despite this reduction, no linear pattern is observed, as some years, such as 2018 and 2019, showed peaks in hospitalizations (435 and 443 cases, respectively). Regarding deaths, the annual number fluctuated between 2 (2019) and 14 (2015), totaling 79 cases during the period. When comparing the proportion of deaths to hospitalizations, it is noted that years like 2017 (9 deaths for 349 hospitalizations) and 2024 (11 deaths for 341 hospitalizations) had higher rates, suggesting variations in lethality over time, as shown in Figure 1.

**Figure 2:**
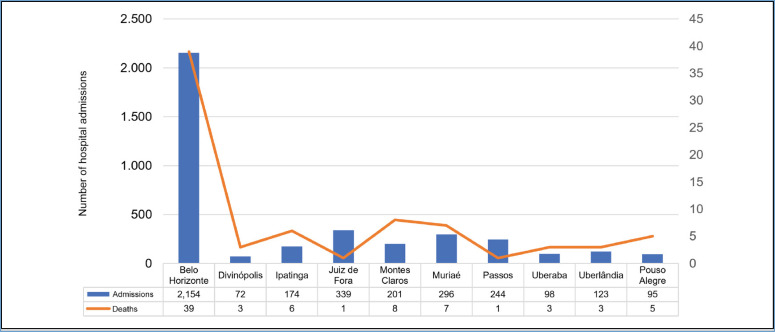
Distribution of the profile of hospitalizations/deaths by municipalities of patients with malignant neoplasms of the bones and articular cartilage in the pediatric population of the State of Minas Gerais, 2015 to 2024.

Source: DATASUS-SIH.

From 2015 to 2024, the municipality of Belo Horizonte concentrated the highest absolute number of hospitalizations for malignant neoplasms of the bones and articular cartilage in the pediatric population of Minas Gerais, with 2,154 records, followed by Juiz de Fora (339 hospitalizations) and Muriaé (296 hospitalizations). Regarding deaths, Belo Horizonte also had the highest absolute number, with 39 records, followed by Montes Claros (8), Muriaé (7), and Ipatinga (6). It is noteworthy that Juiz de Fora, despite having a high number of hospitalizations, recorded only one death during the period, as shown in Figure 2.

Table 1 shows the distribution of the profile (gender, age group, and race) of hospitalizations for malignant neoplasms of the bones and cartilage.

**Table 1:** Profile of pediatric hospitalizations for malignant neoplasms of the bones and articular cartilage in the State of Minas Gerais, 2015 to 2024. (n=3,883).

Year	2015	2016	2017	2018	2019	2020	2021	2022	2023	2024	Total	%
**Hospitalizations by Gender**												
Male	254	250	196	228	228	232	219	207	207	221	2242	57.7
Female	248	205	153	207	215	143	93	128	129	120	1641	42.3
Total	502	455	349	435	443	375	312	335	336	341	3883	100
**Hospitalizations by Age Group**												
Under 1 year	0	1	2	4	19	5	4	0	4	1	40	1.0
1 to 4 years	18	14	2	7	12	9	18	36	20	16	152	3.9
5 to 9 years	98	72	66	36	41	68	24	28	64	79	576	14.8
10 to 14 years	212	188	121	224	204	171	157	153	129	123	1682	43.3
15 to 19 years	174	180	158	164	167	122	109	118	119	122	1433	36.9
Total	502	455	349	435	443	375	312	335	336	341	3883	100
**Hospitalizations by Color/Race**												
White	140	158	113	108	104	109	99	101	101	108	1141	29.4
Black	27	19	20	62	56	28	18	8	29	36	303	7.8
Brown	290	247	184	247	268	165	171	198	191	194	2155	55.5
Yellow	3	2	0	0	0	19	0	0	9	3	36	0.9
Indigenous	14	13	5	1	0	0	1	7	0	0	41	1.1
No Information	28	16	27	17	15	54	23	21	6	0	207	5.3
Total	502	455	349	435	443	375	312	335	336	341	3883	100

Source: DATASUS-SIH.

There is a predominance of the male sex, with 2,242 hospitalizations (57.7%), compared to 1,641 for the female sex (42.3%). When evaluating the distribution by age group, it is noted that the highest concentration of hospitalizations occurred among adolescents aged 10 to 14 years, totaling 1,682 cases (43.3%), followed by the group aged 15 to 19 years, with 1,433 hospitalizations (36.9%). The analysis by color/race indicates a predominance of hospitalizations among brown patients, with 2,155 notifications (55.5%), as shown in Table 1.

Table 2 shows the profile (sex, age group, and race) of deaths due to malignant neoplasms of the bones and articular cartilage in the pediatric population.

**Table 2:** Distribution of the profile of deaths of patients with malignant neoplasms of the bones and articular cartilage in the pediatric population of the State of Minas Gerais, 2015 to 2024. (n=79).

Year	2015	2016	2017	2018	2019	2020	2021	2022	2023	2024	Total	%
**Deaths by Sex**												
Male	7	8	6	5	2	2	2	3	5	7	47	59.5
Female	7	3	3	5	-	2	5	1	2	4	32	40.5
Total	14	11	9	10	2	4	7	4	7	11	79	100
**Deaths by Age Group**												
1 to 4 years	1	1	0	0	0	0	0	0	0	0	2	2.5
5 to 9 years	1	0	0	1	0	1	0	2	1	1	7	8.9
10 to 14 years	4	4	3	2	0	1	2	2	3	3	24	30.4
15 to 19 years	8	6	6	7	2	2	5	0	3	7	46	58.2
Total	14	11	9	10	2	4	7	4	7	11	7	100
**Deaths by Color/Race**												
White	6	3	4	1	1	0	2	2	1	3	23	29.1
Black	2	1	0	1	0	0	0	0	1	0	5	6.3
Brown	6	7	3	7	1	4	4	2	5	8	47	59.5
Indigenous	0	0	1	0	0	0	0	0	0	0	1	1.3
No Information	0	0	1	1	0	0	1	0	0	0	3	3.8
Total	14	11	9	10	2	4	7	4	7	11	79	100

Source: DATASUS-SIH.

From 2015 to 2024, there were 79 deaths due to malignant neoplasms of the bones and articular cartilage in the pediatric population of the State of Minas Gerais. The distribution of deaths by sex revealed a higher frequency among male patients, with 47 cases (59.5%), compared to 32 deaths in female patients (40.5%). This male predominance persisted throughout much of the historical series, with average mortality rates of 2.09% for males and 2.34% for females, values that are close but indicate a slight proportional increase in female patients, despite the lower absolute number of cases. The analysis by age group showed that most deaths occurred among adolescents aged 15 to 19 years (58.2%), followed by the group aged 10 to 14 years (30.4%). Regarding the distribution by color/race, there was a predominance of deaths among brown patients (59.5%), as shown in Table 2.

In financial terms, the average total cost of hospitalizations was R$ 310,272.64 for males and R$ 236,174.59 for females, suggesting higher average costs for male patients. Regarding the average cost by age group, the highest value was observed among adolescents aged 10 to 14 years (R$ 225,912.43), closely followed by the group of 15 to 19 years (R$ 225,851.22), which may be associated with the complexity of treatment in this age range. As for the average hospital stay by gender, female patients stayed hospitalized longer (6.39 days) than male patients (5.27 days). Considering the age group, the highest average was recorded among children under 1 year old (9.25 days), possibly related to greater clinical fragility and the need for intensive care. The other age groups showed averages ranging from 5.39 days (5 to 9 years) to 5.98 days (15 to 19 years), as shown in Table 3.

**Table 3:** Distribution of the average value of expenses and the average length of hospital stay by sex and age group of hospitalizations of patients with malignant neoplasms of the bones and articular cartilage in the pediatric population of the State of Minas Gerais, 2015 to 2024.

Year	Average
**Total Value by Sex**	
Male	310,272,64
Female	236,174,59
Total	546,447,23
**Total Value by Age Group**	
Under 1 year	10,964,634
1 to 4 years	1,816,383,8
5 to 9 years	655,551,14
10 to 14 years	225,912,429
15 to 19 years	225,851,216
Total	546,447,23
**Average Length of Stay by Gender**	**Average**
Male	5.27
Female	6.39
Total	5.75
**Average Length of Stay by Age Group**	**Average**
Under 1 year	9.25
1 to 4 years	5.52
5 to 9 years	5.39
10 to 14 years	5.56
15 to 19 years	5.98
Total	5.75

Source: DATASUS-SIH.

## DISCUSSION

The analysis of hospitalizations for malignant neoplasms of the bones and articular cartilages in Minas Gerais between 2015 and 2024 revealed a general trend of declining hospitalizations, though with annual fluctuations. This pattern finds partial parallels in national and international findings.

In Brazil, Rinaldi da Cruz et al.^11^, analyzing 70,322 pediatric hospitalizations between 2018 and 2023, observed similar annual fluctuations, attributing them to regional inequalities in access, weaknesses in the oncological network, and the impact of the COVID-19 pandemic.

However, unlike the context in Minas Gerais, at the national level, an increase in hospitalizations was observed until 2022, highlighting the heterogeneity among states and suggesting that the reduction observed in Minas may be related to local factors, such as regional health policies, greater centralization of oncological care, or even underreporting during certain periods^11^.

Additionally, Moreira et al.^12^, in the Southeast region, identified annual fluctuations, but with maintenance of clinical severity, as reflected in the relatively stable mortality rate, reinforcing that diagnostic delays and unequal access to specialized therapies remain structural barriers to the proportional reduction of deaths.

In the international context, Helenius and Krieg^13^, when analyzing trends in European countries, highlighted that reductions in hospitalizations do not necessarily imply lower clinical severity, as delays in diagnosis and difficulty in early recognition of symptoms contribute to some patients reaching specialized services at advanced stages of the disease. These findings directly relate to the results from Minas Gerais, where the decline in hospitalizations was not accompanied by a significant reduction in deaths, suggesting the persistence of structural challenges in pediatric oncological care.

Accurate diagnostic evaluation is central to the management of these patients. In this context, Gassert et al.^14^ demonstrated that specific characteristics in computed tomography (CT) and magnetic resonance imaging (MRI), such as intraosseous edema, extraosseous component, endosteal contour, cortical penetration, bone expansion, and presence of limited calcifications, significantly increase the likelihood of atypical cartilaginous tumors (ACTs), reinforcing the need for biopsy or surgical intervention in selected cases.

The identification of these characteristics in long bones allows differentiation between benign tumors, such as enchondromas, and ACTs, contributing to more targeted management and avoiding unnecessary procedures. In Minas Gerais, the centralization of cases in large centers, such as Belo Horizonte and Juiz de Fora, may facilitate the application of these advanced imaging criteria, helping to explain the higher hospital costs and prolonged stays observed in certain groups.

In parallel, in the management of soft tissue tumors containing fat in children and adolescents, Cardoen et al.^15^ showed that the vast majority of lesions are benign and that the indication for biopsy should be guided by factors such as age ≥10 years, genetic predisposition, low fat content, and the presence of myxoid areas. This approach minimizes invasive procedures and general anesthesia, promoting safer, more appropriate management for the pediatric population. These findings complement the discussion on the centralization of care and resource optimization, reinforcing the need for differentiated protocols for children and adolescents, especially in reference centers, and aligning with observations of longer hospital stays and higher costs in complex cases.

The study of the geographic profile of hospitalizations in Minas Gerais shows that Belo Horizonte concentrated the highest absolute number of hospitalizations and deaths, confirming the central role of large reference centers. A similar pattern was described by Moreira et al.^12^ in São Paulo and by Rinaldi da Cruz et al.^11^ in Brazil, with a concentration of cases in the Southeast Region. In Minas Gerais, the case of Juiz de Fora stands out: it had a high volume of hospitalizations but only one death in the historical series, possibly reflecting earlier diagnosis or greater therapeutic efficacy, in contrast to Belo Horizonte, where a higher concentration of cases coincided with higher mortality.

Centralization is also reflected in hospital costs, with Belo Horizonte having the highest total hospitalization costs, followed by Ipatinga and Divinópolis, a pattern consistent with the complexity of services offered in the state. National comparisons, such as those by Moura et al.^16^ in Piauí, show lower average costs, while international studies, such as Fang et al.^17^, indicate that resource limitations in low- and middle-income countries impact both costs and survival, widening inequalities in oncological care.

The demographic profile of hospitalizations in Minas Gerais reveals a male predominance, stable throughout the historical series, consistent with Moreira et al.^12^ and Petca et al.^18^ in Romania, as well as in multicenter cohorts like Kadhim et al.^19^. However, Moura et al.^16^ observed a female predominance in Piauí, indicating that regional, cultural, and social factors may alter the clinical trajectory. Regarding age, adolescents aged 10 to 14 years represented the largest share of hospitalizations in Minas Gerais, aligning with the findings of Rinaldi da Cruz et al.^11^ in Brazil and Moreira et al.^12^ in the Southeast, as well as international studies like Breden et al.^20^, which confirm the disease's preference for the second decade of life.

In relation to race/color, brown patients predominated, reflecting the state's demographic composition, contrasting with the white predominance in the Southeast and approaching the pattern in Piauí; access barriers and socioeconomic vulnerabilities may contribute to this heterogeneity, in line with Helenius and Krieg^13^.

Regarding mortality, Minas Gerais recorded 79 pediatric deaths, with an absolute male predominance and slightly higher average rates among girls, a pattern similar to the national one described by Rinaldi da Cruz et al.^11^ and the regional one by Moreira et al.^12^, but divergent from Piauí, where Moura et al.^16^ recorded a higher female participation. In terms of age, deaths concentrated between 15 and 19 years, with an increase also in children aged 1 to 4 years, aligning with the bimodal pattern described by Moreira et al.^12^ and the international literature of Breden et al.^20^. Regarding race/color, a predominance of deaths among brown individuals was observed, approaching that of Piauí and contrasting with the Southeast, while high rates among indigenous and black individuals indicate the impact of socioeconomic vulnerabilities on clinical outcomes.

The analysis of costs and hospital stays reinforces the influence of sex, age group, and treatment complexity on hospital outcomes. In Minas Gerais, female patients stayed hospitalized longer than males, and those under one year had a higher average length of stay, reflecting greater clinical fragility and the need for intensive care. The 10-14 age group had the highest average costs, possibly due to therapeutic complexity. These findings partially converge with Rinaldi da Cruz et al.^11^ and Moreira et al.^12^ but diverge from Moura et al.^16^, who observed lower costs and less difference between sexes, highlighting the influence of the availability of therapeutic resources. International studies, such as Helenius and Krieg^13^ and Fang et al.^17^, corroborate the relationship between age, clinical complexity, and prolonged hospital stay, while Petca et al.^18^ indicate that complications and diagnostic delays contribute to longer hospitalization times in girls, consistent with the situation in Minas Gerais.

In summary, the findings from Minas Gerais align with patterns observed nationally and internationally regarding the centralization of cases in large centers, male predominance, greater vulnerability of adolescents, and high hospital costs. The integration of findings from Gassert et al.^14^ and Cardoen et al.^15^ reinforces the need for accurate diagnostic evaluation and management based on specific criteria for each tumor type, whether bone or soft tissue, thereby promoting safer therapeutic decisions, avoiding unnecessary biopsies, and enabling more efficient allocation of hospital resources.

However, local divergences in the state of Minas Gerais regarding sex, race/color, and costs highlight the relevance of regional, demographic, and structural factors. The body of evidence reinforces that timely screening policies, shorter time to specialized care, and increased access to high-complexity therapies are essential to reduce lethality among the most vulnerable groups, improve hospital outcomes, and promote equity in pediatric oncological care.

## CONCLUSION

The study allowed for the characterization of the profile of deaths due to malignant neoplasms of bones and articular cartilage in the pediatric population of Minas Gerais between 2015 and 2024. A trend toward reduced hospitalizations was observed, but mortality remained stable, reflecting challenges in early detection and access to specialized therapies. Belo Horizonte concentrated the highest number of cases and deaths, while other municipalities, such as Juiz de Fora, showed a high frequency of hospitalizations but low mortality. Male patients predominated, particularly adolescents aged 10 to 19 years and of mixed race. Younger patients and females had longer hospital stays, and costs were higher among adolescents aged 10 to 14 years. The findings reinforce the need for early detection policies, increased access to specialized oncological care, and the reduction of regional inequalities, aiming to improve clinical outcomes and promote equity in pediatric care.

## Data Availability

The underlying contents of the research text are contained in the manuscript.
